# Adaptive Interlimb Coordination Mechanism for Hexapod Locomotion Based on Active Load Sensing

**DOI:** 10.3389/fnbot.2022.645683

**Published:** 2022-02-08

**Authors:** Akira Fukuhara, Wataru Suda, Takeshi Kano, Ryo Kobayashi, Akio Ishiguro

**Affiliations:** ^1^Research Institute of Electrical Communication, Tohoku University, Sendai, Japan; ^2^Program of Mathematical and Life Sciences, Graduate School of Integrated Sciences for Life, Hiroshima University, Hiroshima, Japan

**Keywords:** hexapod locomotion, inter-limb coordination, decentralized control algorithm, active load sensing, chains of reflex

## Abstract

Insects can flexibly coordinate their limbs to adapt to various locomotor conditions, e.g., complex environments, changes in locomotion speed, and leg amputation. An interesting aspect of insect locomotion is that the gait patterns are not necessarily stereotypical but are often highly variable, e.g., searching behavior to obtain stable footholds in complex environments. Several previous studies have focused on the mechanism for the emergence of variable limb coordination patterns. However, the proposed mechanisms are complicated and the essential mechanism underlying insect locomotion remains elusive. To address this issue, we proposed a simple mathematical model for the mechanism of variable interlimb coordination in insect locomotion. The key idea of the proposed model is “decentralized active load sensing,” wherein each limb actively moves and detects the reaction force from the ground to judge whether it plays a pivotal role in maintaining the steady support polygon. Based on active load sensing, each limb stays in the stance phase when the limb is necessary for body support. To evaluate the proposed model, we conducted simulation experiments using a hexapod robot. The results showed that the proposed simple mechanism allows the hexapod robot to exhibit typical gait patterns in response to the locomotion speed. Furthermore, the proposed mechanism improves the adaptability of the hexapod robot for leg amputations and lack of footholds by changing each limb's walking and searching behavior in a decentralized manner based on the physical interaction between the body and the environment.

## 1. Introduction

Insects exhibit versatile interlimb coordination patterns to move around adaptively. For example, some insects possess various gait patterns (e.g., wave gait, tetrapod gait, and tripod gait) that change in response to changes in locomotion speed and loads applied to the body (Wilson, 1966; Dean, 1991; Zollikofer, 1994; Wosnitza et al., 2013). Furthermore, they can generate feasible locomotor patterns in response to leg amputation (Hughes, 1957; Delcomyn, 1991; Grabowska et al., 2012). In addition to steady walking, they flexibly change limb motion between walking and searching steps when they walk on uneven terrain with some gaps in the foothold (Pearson and Franklin, 1984; Theunissen and Dürr, 2013; Theunissen et al., 2014, 2015). While insects exhibit long stride steps during steady walking (i.e., walking) on uneven terrain, they exhibit short searching steps where the limb repeats a retracting and protracting motion in a short stride distance to obtain secure footholds. Understanding these flexible interlimb coordination mechanisms underlying insect adaptive walking sheds new light on developing adaptive legged robots that can move around stably through rough environments (e.g., disaster sites).

Biological and modeling studies have investigated decentralized control mechanisms underlying adaptive insect locomotion through comparative studies focusing on distinct insect species, e.g., stick insects and cockroaches (Ayali et al., 2015a). Stick insects (e.g., *Phasmida*) can climb unpredictable environments, and their relatively slow locomotor patterns allow researchers to address underlying sensory-motor mechanisms. Biological studies have elucidated that thoracic neural circuits generate rhythmic locomotor patterns neither sensory input from the leg nor the descending command from the brain (Mantziaris et al., 2020). Furthermore, sensory input in the limbs contributes to modifying intra- and interlimb coordination for adaptive stick insect locomotion. In contrast to stick insects, cockroaches (e.g., *Blattaria*) exhibit fast and stable locomotion and are ideal insects to address the interaction between neural control and body dynamics. While their conservative tripod gait patterns are generated by CPG, their flexible body can negotiate uneven terrains (Full et al., 1998; Watson et al., 2002; Weihmann et al., 2017). Furthermore, recent studies elucidate the sensory feedback mechanism underlying cockroach‘s locomotion in which signals from mechanoreceptors modulate muscle contractions to establish interlimb coordination (Ayali et al., 2015b; Weihmann et al., 2017). Although the mathematical models for insect‘s interlimb coordination have been developed differently depending on the focusing insect animals (e.g., stick insects and cockroaches), the common distributed control mechanisms in distinct insect animals have induced to unify them into common limb coordination model (Koditschek et al., 2004; Büschges et al., 2008; Daun-Gruhn, 2011; Toth et al., 2013). However, these unified models are too complex to analyze and apply to legged robots in simple manners.

In contrast to complex models describing the insect‘s sensory-motor system with large numbers of differential equations, redacted models significantly help us test hypotheses and interpret the substantial interlimb coordination mechanism underlying insect locomotion (Kimura et al., 1993; Dürr et al., 2004; Kukillaya et al., 2009; Owaki et al., 2017). The simple models reduced dimensions by using simple elementary processes, e.g., phase oscillators and reflexes, to generate interlimb coordination. For example, Cruse et al. proposed a series of reflex rules based on the behaviors of stick insects (Dürr et al., 2004). They predicted the pathway of sensory-motor modulation for interlimb coordination in the insect animal's thoracic nervous system. For another example, Owaki et al. proposed a simple CPG model where one phase oscillator controls each limb's stride motion and demonstrated that phase modulations depending on loads of limbs contribute to generating various gaits locomotion speed and leg amputation (Owaki et al., 2017). Regarding adaption to uneven environments, however, previous models still require recruiting a large number of neural components for modulating interlimb coordination depending on situations (Durr, 2001; Bläsing, 2006; Schilling et al., 2013a,b; Ngamkajornwiwat et al., 2020). This is because the limb without a stable foothold should adaptively change its foot trajectory and frequency comparing other limbs to search steady footholds. Therefore, the development of a simple interlimb coordination mechanism involving searching behavior will contribute to deeply understanding the essential mechanism underlying flexible insect locomotion.

To this end, this study develops a simple interlimb coordination model to extract substantial mechanisms underlying adaptive hexapod locomotion, including searching behavior on uneven terrain. We hypothesize that a simple local sensory feedback mechanism, “active load sensing,” plays an essential role in generating flexible hexapod interlimb coordination patterns in flat and uneven environments. In this scheme, each limb actively moves and detects the reaction force from the ground to judge whether it plays a pivotal role in maintaining the steady support polygon. As a result of the simulation experiments, a hexapod robot that could generate flexible gait patterns in response to locomotor speed and leg amputation was developed. Furthermore, the robot flexibly changed its limb behaviors between the walking step in steady walking and the searching step depending on the lack of the foothold. During particular limb searching, other limbs flexibly modulate their interlimb coordination through the same mechanism in walking in flat terrain. These results suggest that a simple decentralized control mechanism exploiting physical interaction between body and environment (e.g., the proposed active load sensing) allows insects to generate flexible interlimb coordination for flat terrain and unpredictable environments.

The remainder of this paper is organized as follows: Section 2 exploits the proposed simple interlimb coordination mechanism; Section 3 presents the results of the 3D simulation; Sections 4 and 5 present the discussion and conclusion.

## 2. Model

According to insect behaviors, the insects adaptively generate long limb strides for walking and short limb strides for searching. Besides, the periods of one limb stride locally and drastically change during a pass through uneven terrains. Therefore, modeling based on phase oscillators is required to discontinuously modulate the phase (e.g., phase reset) and also modulate limb trajectories, resulting in a complex interlimb coordination mechanism. To develop a simple model, this study employs two feedforward limb control modes and four fundamental transition rules that induce a hexapod robot to generate walking and searching behaviors. In the following modeling section, we first explain a robot model in the simulation environments. Then, we illustrated two basic limb control modes “*swing mode*” and “*stance mode*” and fundamental transition rules.

**Figure 1:**
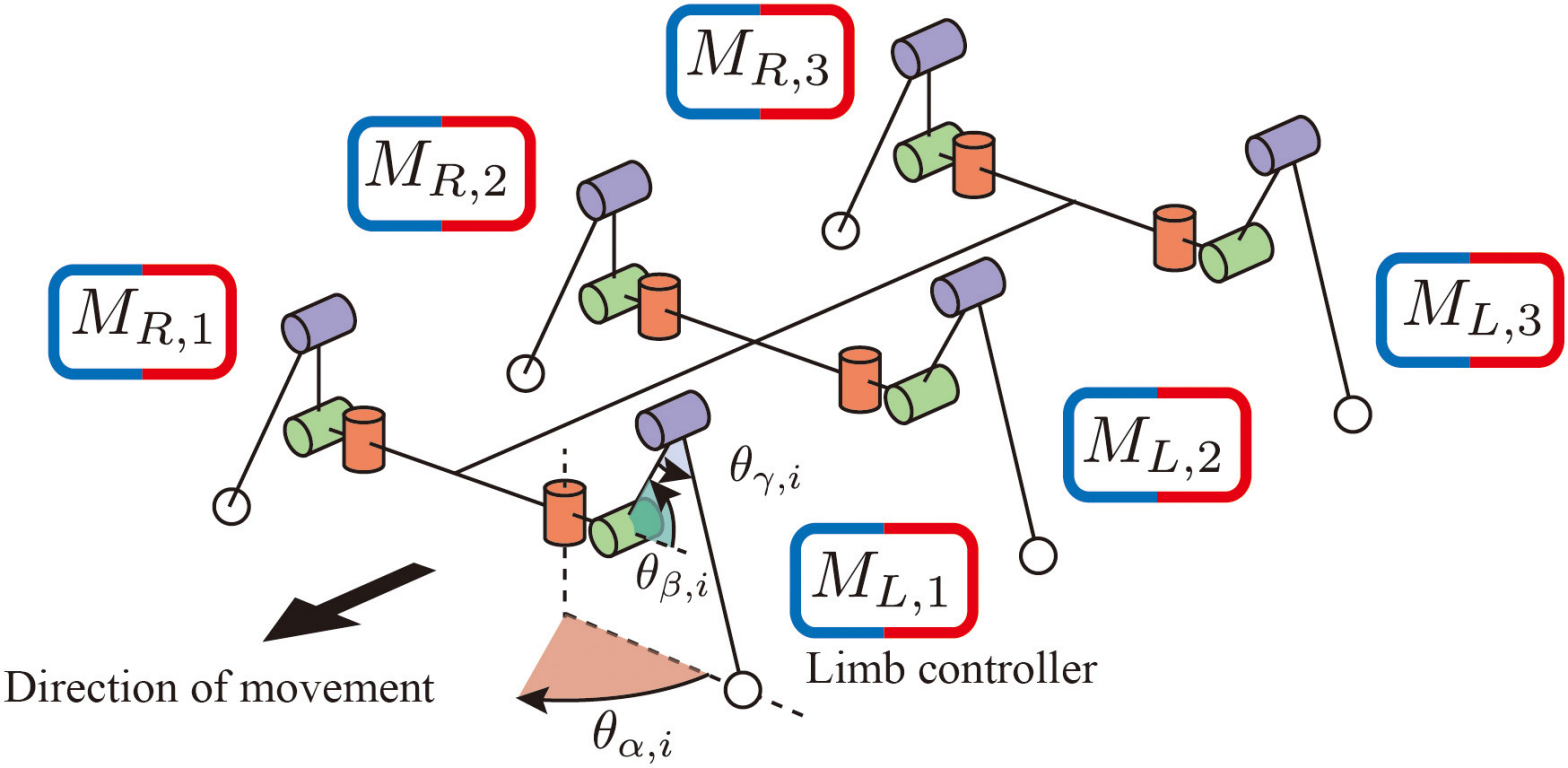
The mechanical structure of the hexapod robot. Each limb has three degrees of freedom. Joint α generates retracting and protracting motions. Joints β and γ generate elevating motions. Each limb has a controller to generate limb motion in a decentralized manner.

Regarding the mechanical structure, a robot consists of six identical limb units and a rigid trunk unit, as shown in Figure 1. Each limb has three degrees of freedom: joint α connects the trunk unit and limb unit and generates the protraction and retraction motion by changing the angle of the joint $\theta_{i,j}^{\alpha }$ (*i* = *R, L* and *j* = 1, 2, 3). The other two joints β and γ generate flexing/extending and elevating motions by changing the angles of the joints $\theta_{i,j}^{\beta }$ and $\theta_{i,j}^{\gamma }$.

**Figure 2:**
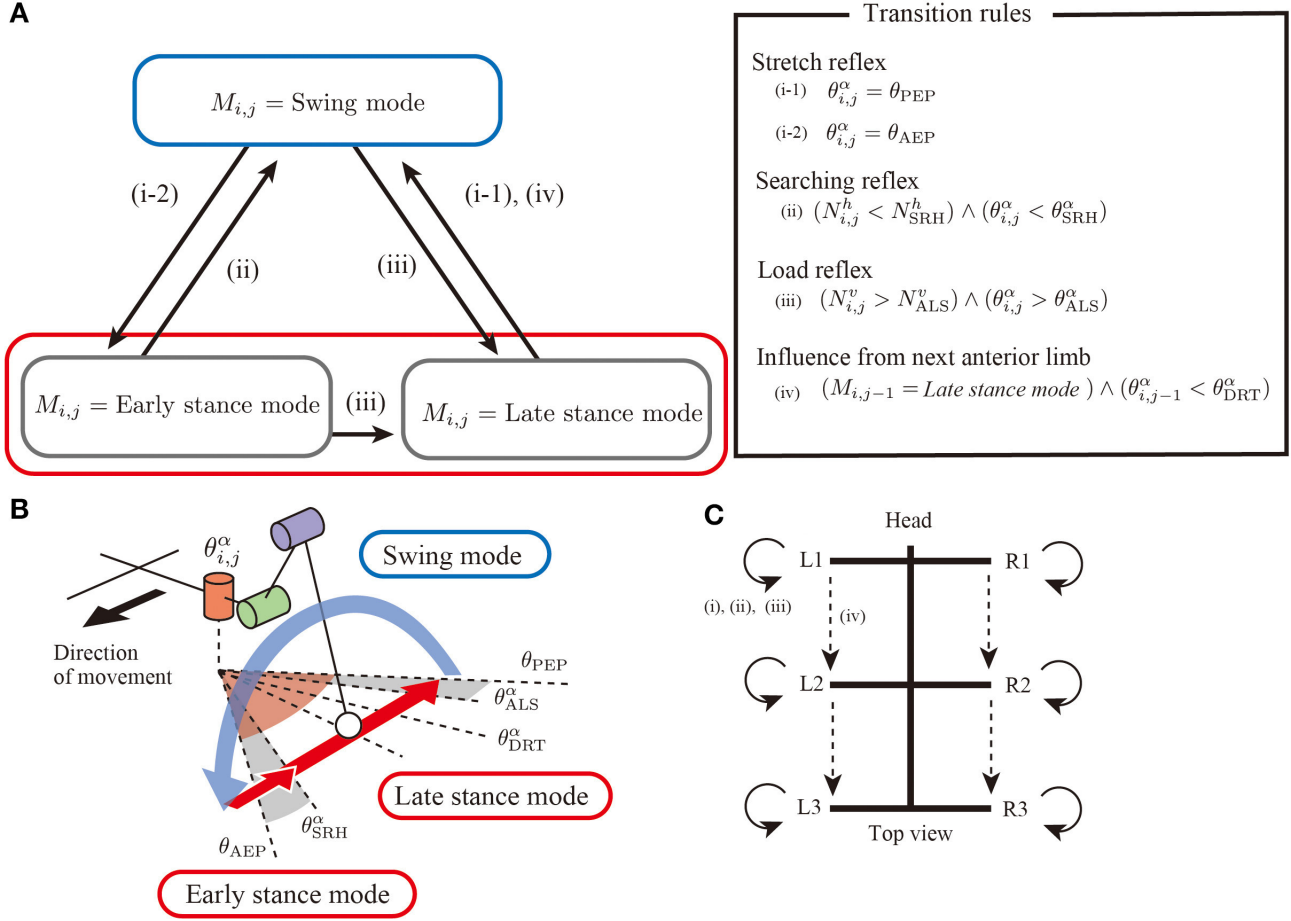
Overview of an interlimb coordination mechanism for hexapod locomotion. **(A)** Two control modes(*swing mode* and *stance mode*) and transition conditions. **(B)** Schematics of threshold values in limb trajectory. **(C)** Neural connectivity between the limbs in the proposed model.

Regarding the basic components of a limb controller, each limb has a controller with two control modes, that is, *stance modes* and *swing mode* to generate limb stride motions, as shown in Figure 2A. The controller state is described with the symbol *M*_*i,j*_. When *M*_*i,j*_ = *Swing mode*, the limb controller is in the swing mode, and the limb generates protracting motion. Furthermore, the proposed model has two stance modes: early stance mode and late stance mode. In both stance modes, the limb generates retracting motion for kicking the ground.

In the proposed robot mode, we simplify the coordination between the joints (i.e., intralimb coordination) to realize a specific foot trajectory. In all control modes, the joint α is controlled to achieve the target joint angular velocity ${\bar{\dot{\theta }}}_{i,j}^{\alpha }$. In the swing mode (*M*_*i,j*_ = *Swing mode*), ${\bar{\dot{\theta }}}_{i,j}^{\alpha }$ is set to a positive constant value ω_sw_ to generate the protracting motion, whereas in the stance modes (*M*_*i,j*_ = *Early stance mode, Late stance mode*), ${\bar{\dot{\theta }}}_{i,j}^{\alpha }$ is a negative constant value ω_st_ to generate the retracting motion. The joints β and γ are controlled to achieve a joint target angle ${\bar{\theta }}_{i,j}^{\beta }$ and ${\bar{\theta }}_{i,j}^{\gamma }$, respectively, so that the foot moves along a specific trajectory, as shown in Figure 2B. Details of the foot trajectory design are described in Appendix A. Note that there is no additional control mode to stand stably (not walk) in the proposed mode. To realize a transition between walking and standing, the target angular velocity of *alpha* joint ${\bar{\theta }}_{i,j}^{\alpha }$ will change between negative for walking and zero for standing. However, in the present study, we focus on flexible changes walking and searching and set the parameter ${\bar{\theta }}_{i,j}^{\alpha }$ constant value for limb stride motion.

To generate adaptive interlimb coordination patterns, each controller should switch the control modes depending on the situation. The present study proposes four simple transition rules between the swing and stance modes, as shown in the overview control scheme (Figure 2C). Note that most rules conduct in a decentralized manner by exploiting physical interactions between the whole body and the environment. The details of the four simple rules are explained in the following sections.

### Rule (i): Stretch Reflex

**Figure 3:**
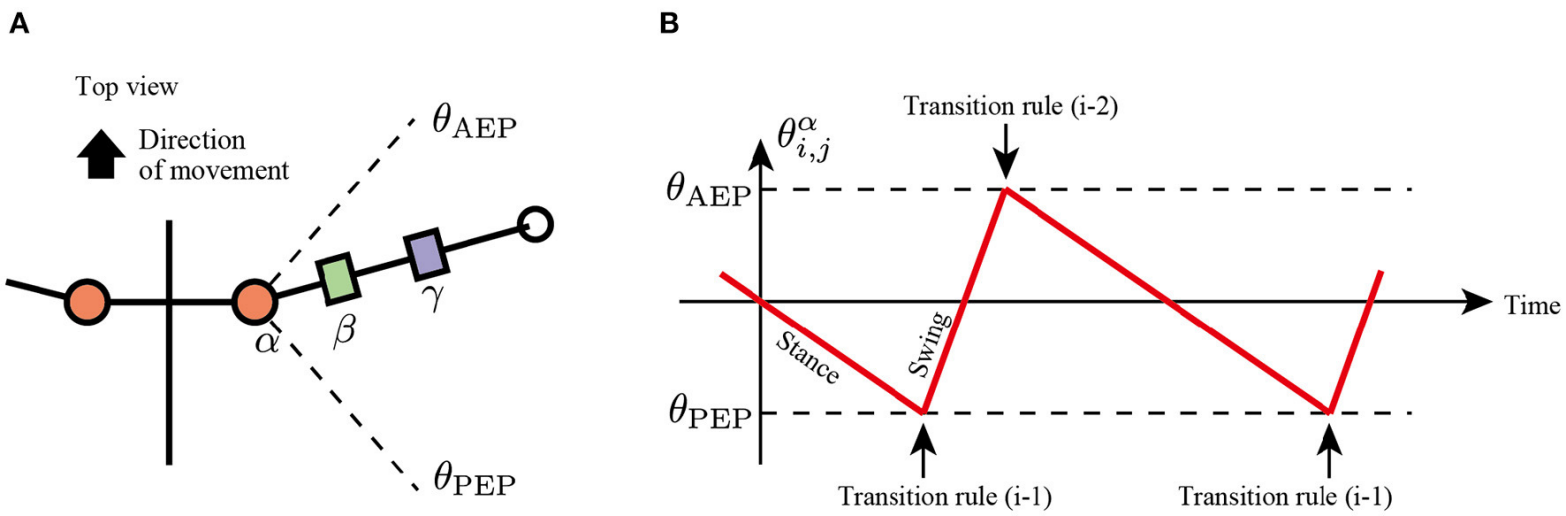
Transition mechanism based on α joint angle, $\theta_{i,j}^{\alpha }$. **(A)** Top view of the robot and threshold joint angles, θ_AEP_ and θ_PEP_ for the anterior extreme position (AEP) and posterior extreme position (PEP) transitions, respectively. **(B)** Example of changes in the control mode *via* transition rule (i).

In the first rule, the limb changes its control modes between the swing and stance mode at the anterior extreme position (AEP) and posterior extreme position (PEP) of the foot to generate periodic limb stride motion (Figure 3). If the angle of joint α in the swing mode reaches a positive threshold angle θ_AEP_ ($\theta_{i,j}^{\alpha }≧\theta_{\text{AEP}}$), then the limb controller changes its mode from swing mode to early stance mode. In contrast, if the joint angle α in the stance mode reaches a negative threshold angle θ_PEP_ ($\theta_{i,j}^{\alpha }≦\theta_{\text{PEP}}$), then the limb controller changes its mode from stance to swing. The above transitions are described as follows:

(1) $$\begin{array}{l} \{\begin{array}{c} \text{if }\theta_{i,j}^{\alpha }≧\theta_{\text{AEP}}\text{ then }M_{i,j}=Swing\;mode\to Early\;stance\;mode,\\ \text{if }\theta_{i,j}^{\alpha }≦\theta_{\text{PEP}}\text{ then }M_{i,j}=Late\;stance\;mode\to Swing\;mode. \end{array} \end{array}$$

Note that after mode transition from the swing to stance at the AEP point, the limb first becomes the early stance mode (*M*_*i,j*_ = *Early stance mode*), not the late stance mode (*M*_*i,j*_ = *Late stance mode*).

### Rule (ii): Searching Reflex

The second rule realizes adaptive switching between stepping and searching behavior depending on the lack of footholds. Although insects usually exhibit long retracting and protracting motions to generate stride lengths, the insect repeats short retracting and protracting motions to search for the next foothold in response to the foothold gaps (Pearson and Franklin, 1984; Theunissen and Dürr, 2013; Theunissen et al., 2014).

**Figure 4:**
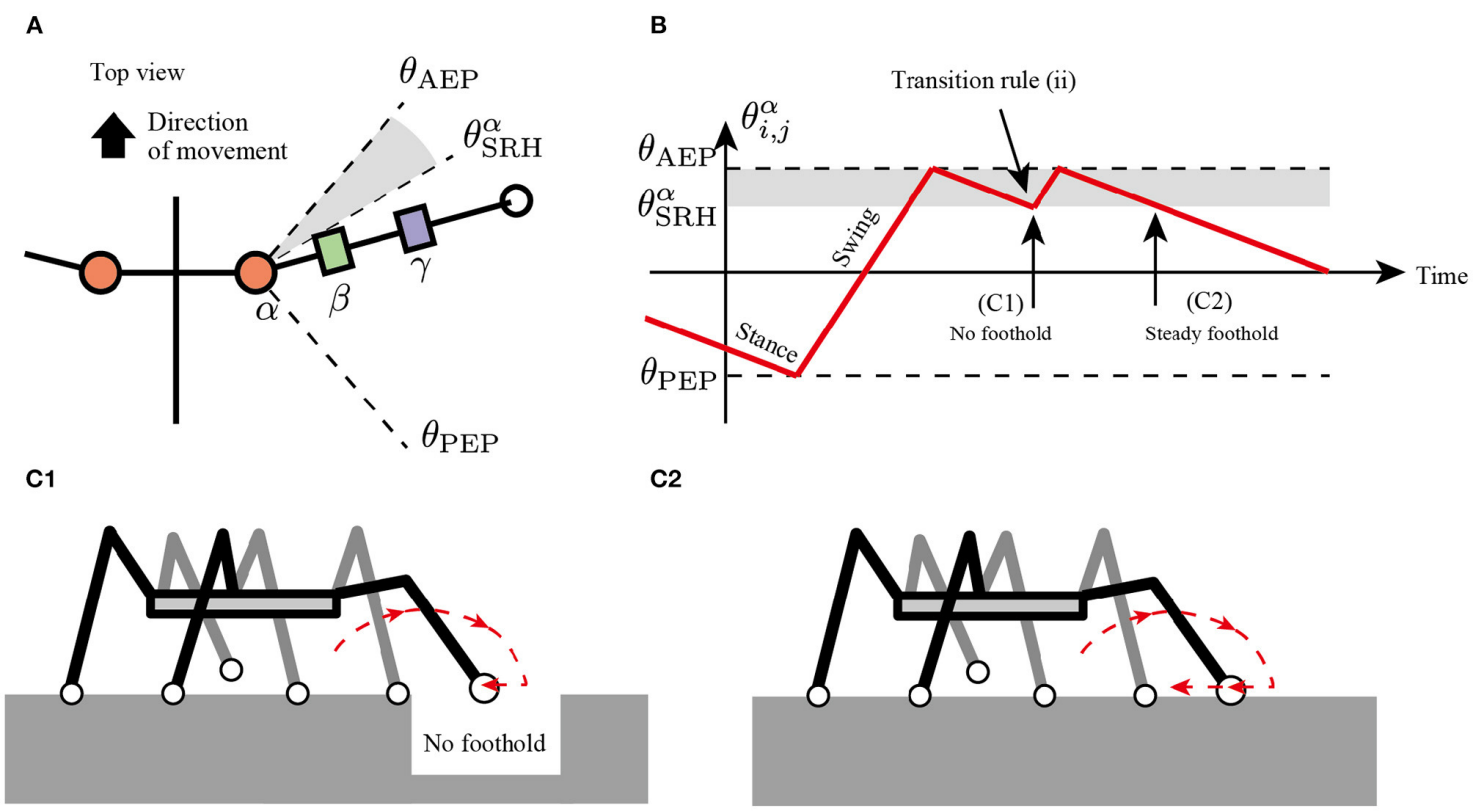
Transition rule (ii) to secure foothold. **(A)** Top view of the body and a range of α joint angles, $\theta_{\text{SRH}}^{\alpha }$. **(B)** Example of changes in control mode *via* transition rule (ii). When $\theta_{AEP}>\theta_{i,j}^{\alpha }>\theta_{\text{SRH}}^{\alpha }$ after the transition in AEP, the robot protracts the limb and tends to kick the ground. **(C1)** If the limb cannot perceive the ground reaction force (GRF), the limb controller changes to the swing mode, resulting in a short step. **(C2)** If the limb successfully kicks the ground, the limb remains in the stance mode, resulting in a long step.

To implement the flexible changes between the stepping and searching behaviors, the present study assumes a simple transition rule for the transition from the stance mode to the swing mode as follows:

(2) $$\begin{array}{l} \text{if }\left(N_{i,j}^{h}<N_{\text{SRH}}^{h}\right)\wedge \left(\theta_{i,j}^{\alpha }<\theta_{\text{SRH}}^{\alpha }\right)\text{then }M_{i,j}\\ =Early\;stance\;mode\to Swing\;mode, \end{array}$$

where $N_{i,j}^{h}$ is a horizontal component of ground reaction force (GRF) applied at the *i, j* limb (driving force is positive), $N_{\text{SRH}}^{h}$ is a threshold value for detecting where the limb obtains the foothold, and $\theta_{\text{SRH}}^{\alpha }$ is a constant value describing a range of joints α for the searching behavior. According to rule (i), the protracted limb changes the control mode from swing to stance and starts to retract. If the limb has no propulsive force after the retraction motion, then the limb changes to the swing mode immediately, resulting in the protracting motion (Figure 4).

### Rule (iii): Active Load Sensing at the Beginning of the Swing Phase

**Figure 5:**
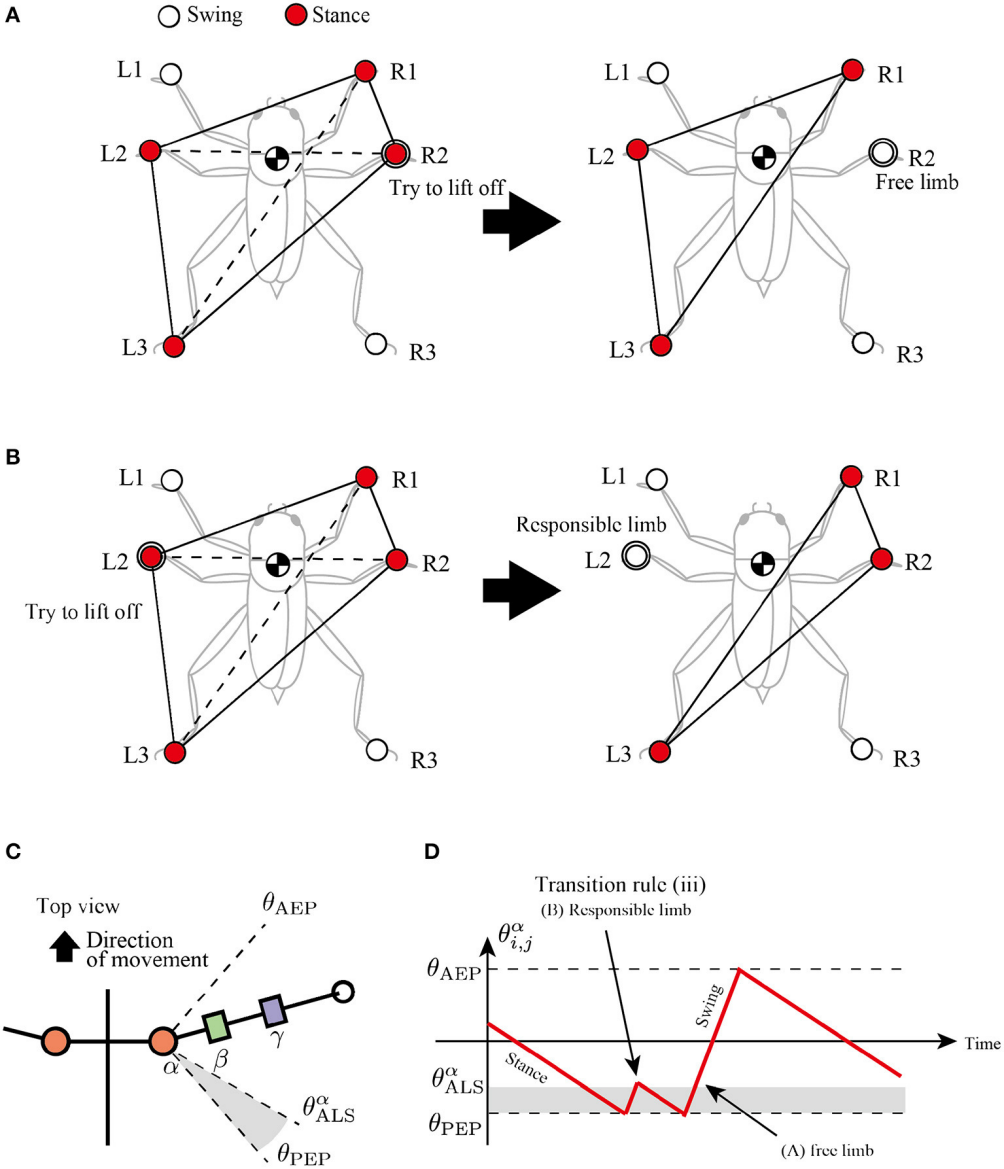
Active load sensing scheme for detecting free and responsible limbs in hexapod locomotion. **(A)** Free limb situation. After the R2 limb lifts, the center of mass (COM) is still in the supporting polygon. **(B)** Responsible limb situation. After the L2 limb lifts, the COM moves outside the support polygon. By exploiting the physical interaction between the body and the environment, each limb can simply modulate its control mode for the steady support polygon. **(C)** Top view of the body and a range of α joint angles, $\theta_{\text{ALS}}^{\alpha }$. **(D)** Example of changes in control mode *via* transition rule (iii).

The third rule is attempted to secure a support polygon at the beginning of the swing phase in a decentralized manner. The support polygon is a convex horizontal region whose vertices correspond to the support limbs. For example, in Figure 5A, the support polygon comprises the contact points R1, R2, L2, and L3. When the center of mass (COM) lies in the support polygon, static stability is achieved during locomotion.

To achieve static stability during locomotion through a decentralized control manner, this study classifies the stance limbs into two types: “free limb” and “responsible limb.” The free limb is a stance limb in which the robot maintains static stability when the concerned limb lifts off the ground. For example, consider the support polygon shown in Figure 5A, where the R1, R2, L2, and L3 limbs are in the stance phase. When the R2 limb lifts off, the new support polygons with R1, L2, and L3 still contain the COM of the insect, maintaining static stability. Therefore, the R2 limb can be classified as a free limb. In contrast to the free limb, the responsible limb is a stance limb in which the robot cannot keep the static polygon when the concerned limb lifts off the ground. For example, in Figure 5B, when the L2 limb lifts off, the COM of the insect is located outside the new support polygon with R1, R2, and L3, resulting in a lack of static stability. Consequently, the L2 limb in Figure 5B can be classified as a responsible limb. For stable and adaptive locomotion, the challenge is to instantly detect the free and responsible limbs and accordingly modulate the limb movements to maintain static stability.

The proposed study distinguishes between free and responsible limbs and modulates the limb control mode adaptively in a simple, decentralized manner. For detection of the limb state, the stance limb close to the PEP first attempts to lift off the ground. If the concerned limb perceives no GRF, then it can be interpreted as a free limb, and it changes the stance mode to swing mode. In contrast, if the concerned limb still perceives GRF, then it can be interpreted as a responsible limb and should be maintained in the stance mode. We describe these sequences of action and detection as “*active load sensing*.”

In the proposed model, we implement active load sensing around the PEP as follows. The lifting action is realized by other transition rules. Then, the sensory feedback mechanism based on active load sensing is described as follows:

(3) $$\begin{array}{l} \text{if }\left(N_{i,j}^{v}≧N_{\text{ALS}}^{v}\right)\wedge \left(\theta_{i,j}^{\alpha }>\theta_{\text{ALS}}^{\alpha }\right)\\ \text{then }M_{i,j}\to Late\;stance\;mode. \end{array}$$

where $N_{i,j}^{v}$ is a vertical component of GRF applied at the *i, j* limb, $N_{\text{ALS}}^{v}$ is a positive constant value for a threshold whether the limb is loaded or unloaded, and and $\theta_{\text{ALS}}^{\alpha }$ is a constant value to describe a blind-sector angle for active load sensing (Figure 5C). When $\theta_{\text{ALS}}^{\alpha }>\theta_{i,j}^{\alpha }>\theta_{\text{PEP}}$ during the swing mode, the limb maintains the protracting motion for lifting. After the lifting motion, if the protracting limb perceives GRF, it changes the control mode from swing to stance immediately like Figure 5D to achieve static stability.

### Rule (iv): Sensory Feedback From Next Anterior Limb

**Figure 6:**
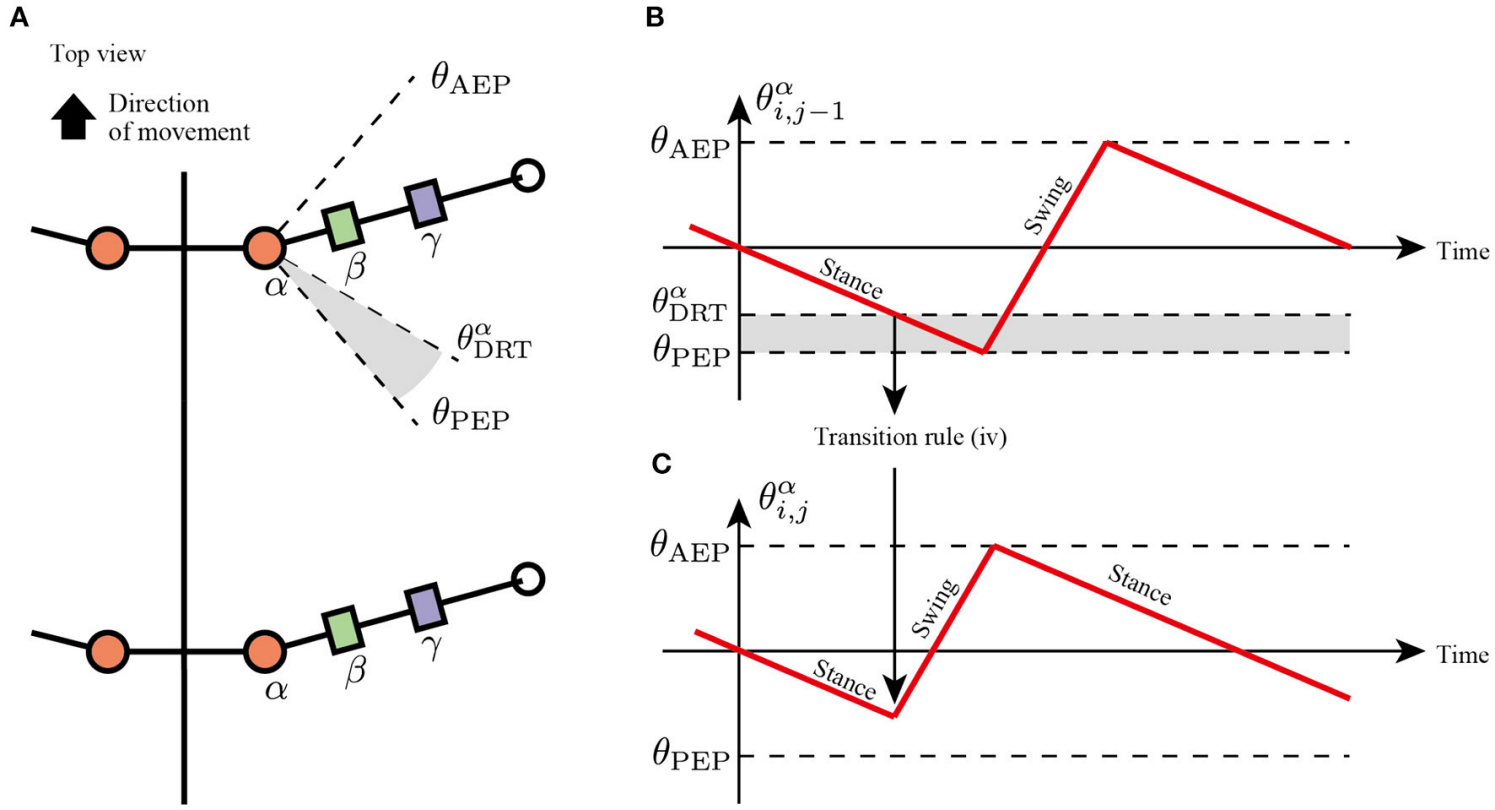
Transition to swing phase depending on the position of next anterior limb. **(A)** Top view of the robot and threshold joint angle $\theta_{\text{DRT}}^{\alpha }$ for the effect from anterior to posterior limbs. **(B)** Changes in joint α of the anterior limb. When $\theta_{i,j-1}^{\alpha }<\theta_{\text{DRT}}^{\alpha }$, the limb sends a signal for the posterior limb to transition from the stance to swing mode. **(C)** Changes in joint α of the posterior limb. The posterior limbs move to the swing mode before reaching the PEP angle θ_PEP_.

In slow insect walking gaits (e.g., tetrapod gait and wave gait), the limbs of the ipsilateral side exhibit a metachronal wave from the tail to the head (i.e., wave gait; Wilson, 1966). Based on the insect walking trend, we assume the fourth transition rule in which each limb tends to switch its control mode from the stance mode to the swing mode when the anterior next limb reaches the PEP (Figure 6). More specifically, the enforcing early protraction refers to whether the angle of the next anterior joint α, $\theta_{i,j-1}^{\alpha }$, achieves a threshold angle $\theta_{\text{DRT}}^{\alpha }$ using the following equation:

(4) $$\begin{array}{l} \text{if }\theta_{i,j-1}^{\alpha }≦\theta_{\text{DRT}}^{\text{α}}\\ \text{then }M_{i,j}=Late\;stance\;mode\to Swing\;mode\;\left(\text{for }j\neq 1\right).\; \end{array}$$

where $\theta_{\text{DRT}}^{\text{α}}$ is the threshold angle in the joint α that detects the limb closer to the PEP. Note that rule (iv) is the only transition rule that assumes the neural coupling between limbs in the proposed model.

## 3. Results

To evaluate the proposed interlimb coordination mechanism, the present study conducts three kinds of simulation experiments: the emergence of typical hexapod locomotion, adaptation to leg amputation, and adaptation to gap environment. We use an open dynamics engine (ODE) to calculate the hexapod robot's three-dimensional physical dynamics in all experiments. The parameters in the simulation are heuristically determined as shown in Table 1 so that the robot can generate a typical tripod gait when the target angular velocities in the stance mode ω_st_ are the same as that in the swing mode ω_sw_.

**Table 1:** Parameters in simulation experiments.

**Body**			**Control**		
**Parameters**	**Unit**	**Values**	**Parameters**	**Unit**	**Values**
total mass	[kg]	0.92	*W*	[m]	0.12
width	[m]	0.24	*H*	[m]	0.07
length	[m]	0.18	θ_AEP_	[rad]	π/6
height	[m]	0.1	θ_PEP_	[rad]	π/6
*L*_UPR_	[m]	0.12	$\theta_{\text{DRT}}^{\alpha }$	[rad]	π/8
*L*_BTM_	[m]	0.12	$\theta_{\text{ALS}}^{\alpha }$	[rad]	7π/60
			$\theta_{\text{SRH}}^{\alpha }$	[rad]	13π/80
			$N_{\text{ALS}}^{v}$	[N]	0.3
			$N_{\text{SRH}}^{h}$	[N]	0.01
			ω_sw_	[rad/s]	π/3
			ω_st_	[rad/s]	π/3, π/6, π/15

### 3.1. Emergence of Typical Hexapod Gait Patterns

The first simulation experiment aims to evaluate how the proposed rules affect the locomotion patterns of the robot in response to various locomotion speeds. Regarding the experimental setup, the robot with an intact body (i.e., no leg amputation) walks on flat terrain. To address the flexibility of the locomotor patterns in response to locomotor speed, we conducted walking experiments with various swing-stance ratios. More specifically, we set constant values of ω_sw_ and ω_*st*_, as shown in Table 1 for various locomotion frequencies. This setup is according that various insects likely maintain duration in the swing phase while they change the various durations in the stance phase (Wosnitza et al., 2013; Reinhardt and Blickhan, 2014; Weihmann et al., 2017; Dürr et al., 2018). As the phase oscillator based CPG models set the intrinsic frequency of periodic limb motion (Owaki et al., 2017), this study simply set limb swing speed of joint α to generate protract and retract motions in the swing and stance modes.

**Figure 7:**
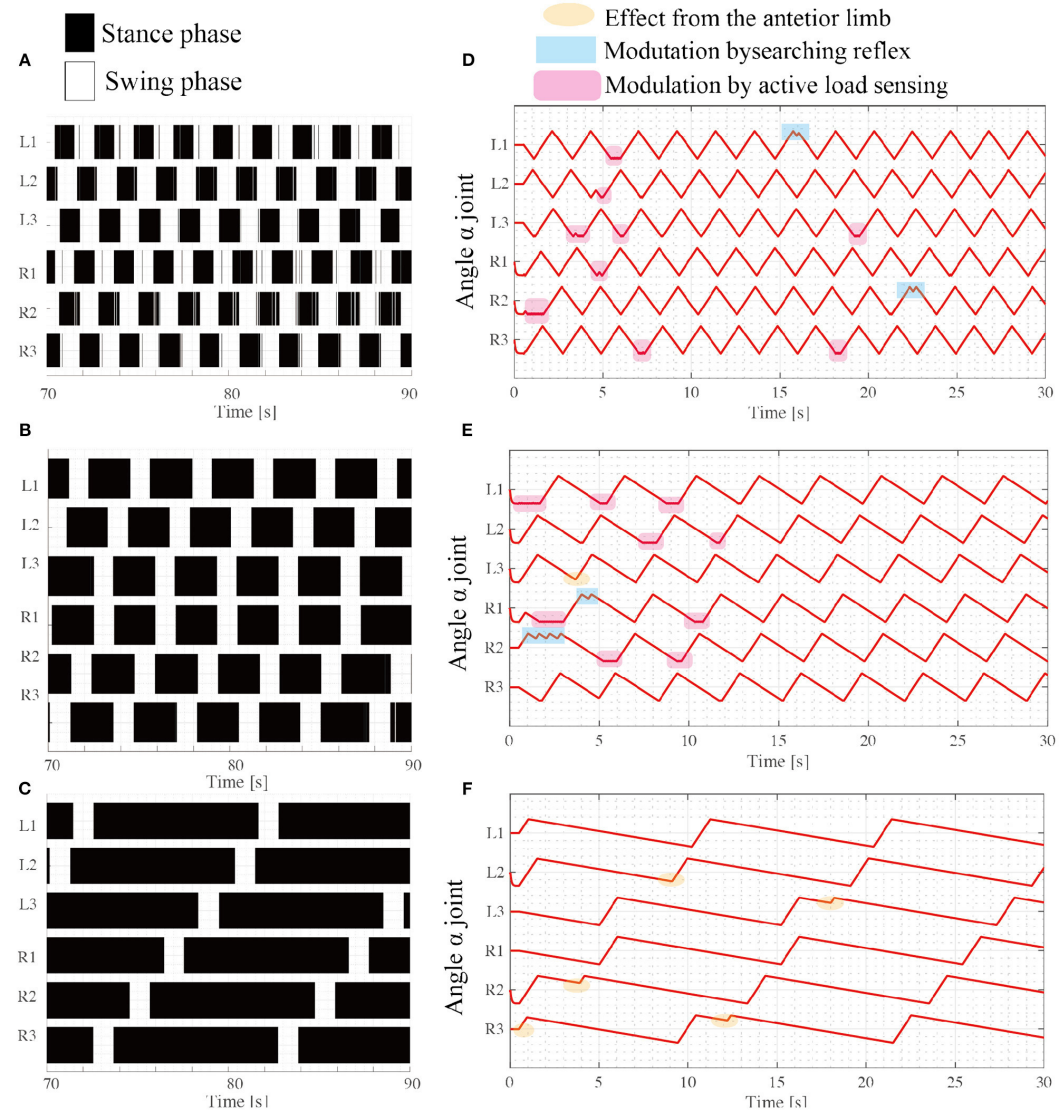
Various insect-like walking patterns depend on the speed ratio during the swing phase. Emerging gait patterns: **(A)** Tripod gait at (ω_sw_, ω_st_) = (π/3, π/3), **(B)** Tetrapod gait at (ω_sw_, ω_st_) = (π/3, π/6), and **(C)** Wave gait at (ω_sw_, ω_st_) = (π/3, π/15). The colored region represents the stance phase where the limb contacts the ground, while the white region represents the swing phase, where the limb has no ground contact. **(D–F)** show changes in the joint angle α from the beginning of the tripod, tetrapod, and wave gaits simulations, respectively.

The results of the simulation experiments showed that the robot exhibited various gait patterns depending on the locomotor speed. When (ω_sw_, ω_st_) = (π/3, π/3), the robot exhibited synchronous coordination in two groups: L1 and R2 are L3 moves in phase, and R1 and L2 are R3 moves in phase as shown in Figure 7A. The interlimb coordination patterns correspond to the tripod gait. The locomotion speed is 10.7 [cm/s]. Additionally, when the target angular velocity in the stance mode ω_st_ decreases to π/6, the robot exhibits different coordination patterns: L1 and R3 synchronize, L2 and R1 synchronize, and L3 and R2 synchronize. These coordination patterns correspond to the typical tetrapod gait, where the two limbs are in the swing phase and the other four limbs support the body weight. The locomotion speed is 8.8 [cm/s]. Furthermore, the parameter ω_st_ decreases to π/15, and the robot exhibits a typical wave gait, as shown in Figure 7C where the ipsilateral anterior limbs move to the swing phase after the next posterior limb. These speed-dependent gait patterns of the robot correspond to the trends of insect locomotor patterns (Wilson, 1966). The locomotion speed is 2.4 [cm/s].

For each locomotor condition, the proposed reflex rules modulate the interlimb coordination patterns as shown in Figures 7D,E. At the beginning of walk, the limb motions are frequently modulated by the reflex rules, for example, active load sensing (rule iii) in Figure 7D. As each interlimb coordination pattern converges, the reflex rules rarely modulate the limb's motion. This is because the locomotor patterns that emerge establish support polygons. With low stance speed (e.g., ω_st_ = π/15), the searching reflex (rule ii) and active load sensing (rule iii) rarely occurs, as shown in Figure 7F, because the long stance period contributes to maintaining the support polygons.

Although the robot exhibits speed dependent interlimb coordination patterns, several limbs show vague takeoff and touchdown, resulting in chattering in the gait diagram in the border between the swing phase and stance phase. This chattering is more conspicuous in a fast walking pattern like tripod gait (Figure 7A) than slow walking gait like metachronal wave gait (Figure 7B). This is because the low duty ratio in fast walking induces difficulty for limbs to translate next supporting polygon. In contrast, the large support polygon in the high duty ratio like Figure 7C facilitates the free limb to translate from the stance mode to the swing mode.

### 3.2. Adaptation to Leg Amputation

The second simulation experiment aims to evaluate the adaptability of the proposed model to leg amputation. In this simulation, we removed the middle limbs (L2 and R2), and the robot walked on leveled ground. We assume that the amputated limb does not induce the next posterior limb to change early from the stance to swing mode, and consequently, rule (iv) is invalidated. Additionally, the control parameters are the same as in the first simulation experiment, as shown in Table 1. Regarding angular velocities, we set (ω_sw_, ω_st_) = (π/3, π/15), referring to the low locomotion speed.

**Figure 8:**
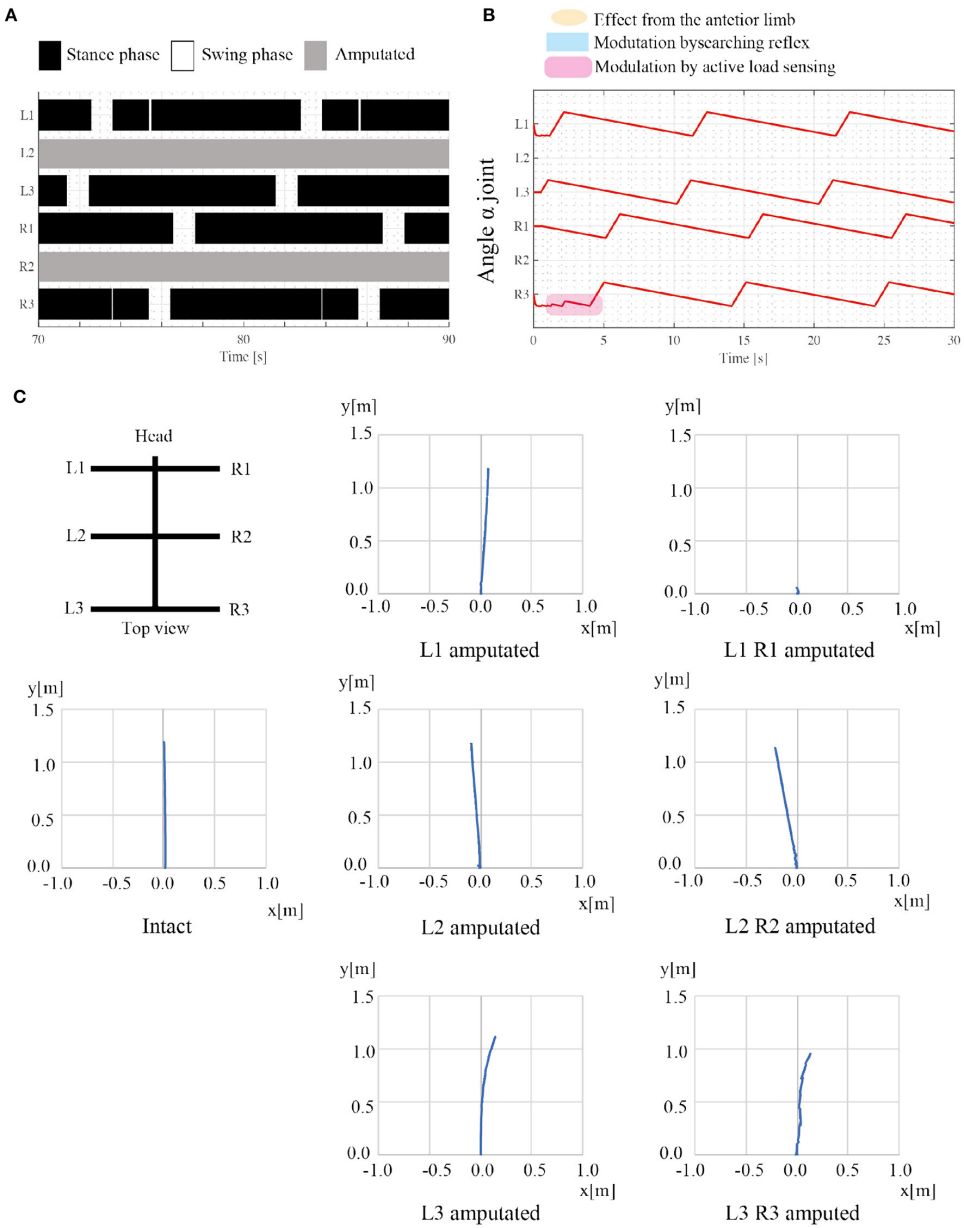
Results of adaptation to leg amputations. **(A)** Gait diagram of the walking robot with amputated L2 and R2 limbs. Regarding the target angular velocity, (ω_sw_, ω_st_) = (π/3, π/15). The robot exhibited a lateral sequence gait. **(B)** The history of each joint angle α from the beginning of walking with the leg amputation. **(C)** Robot trajectories with various combinations of leg amputation. The robot can move despite the leg amputations, whereas the robot stacks when the L1 and R1 limbs are amputated. Note that there is no direction control mechanism and the direction of robot movement changes depending on the physical interaction between the robot and the environment.

Figure 8 shows the results of the amputation. When L2 and R2 limbs are amputated, the robot generates feasible locomotor patterns that differ from the locomotor patterns by the intact robot. As shown in Figure 8A, the posterior limb on the ipsilateral side (e.g., L3 limb) moves before the anterior limb (e.g., L1 limb) despite no neural communication by the transition rule (iv). Figure 8B shows that the active load sensing (rule (iii)) modulates the responsible limb motions so as to generate feasible interlimb coordination at the beginning of walking. The emerging interlimb coordination well reproduces the actual amputated insect (Hughes, 1957; Graham, 1977; Dean, 1991; Grabowska et al., 2012). Furthermore, Figure 8C shows the trajectory of the walking robot with various combinations of leg amputation at specific periods. The robot can adapt to various combinations of leg amputations (e.g., middle limbs and hind limbs). However, when the L1 and R1 limbs are amputated, the robot falls forward, and it cannot generate feasible locomotor patterns. In the falling case, the COM moves the outside of the support polygon during the stance phase of the middle limbs. Although the proposed model still has room for improvement, it well reproduces parts of the insects' adaptive behavior (e.g., lateral sequence gait with L2R2 amputation) as well as the previous model (Owaki et al., 2017).

### 3.3. Adaptation to Gap Environment

**Figure 9:**
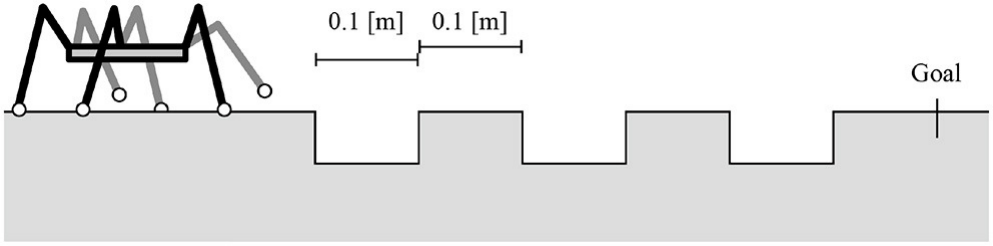
Experimental setup for locomotion on uneven terrain with gaps.

The third experiment addresses the flexible transition between the stepping and searching behaviors in response to the lack of footholds. In this experiment, the robot walked on the ground with gaps and footholds of a specific width, as shown in Figure 9. To evaluate the effect of transition rule (ii), we compare the robot with and without transition rule (ii) and measure the success ratio over 30 trials for the two control conditions. In each trial, the initial joint α angles $\theta_{i,j}^{\alpha }$ are randomly set. When the transition rule (ii) is eliminated, the transition rule (i) at the AEP in Equation (1) is modulated as follows:

(5) $$\begin{array}{l} \begin{array}{l} \text{if }\theta_{i,j}^{\alpha }≧\theta_{\text{AEP}}\text{ then }M_{i,j}=Swing\;mode\to Late\;stance\;mode. \end{array} \end{array}$$

Because of this modulation, the controller has two states: *M*_*i,j*_ = 0 for the swing mode and *M*_*i,j*_ = 1 for the stance mode.

**Figure 10:**
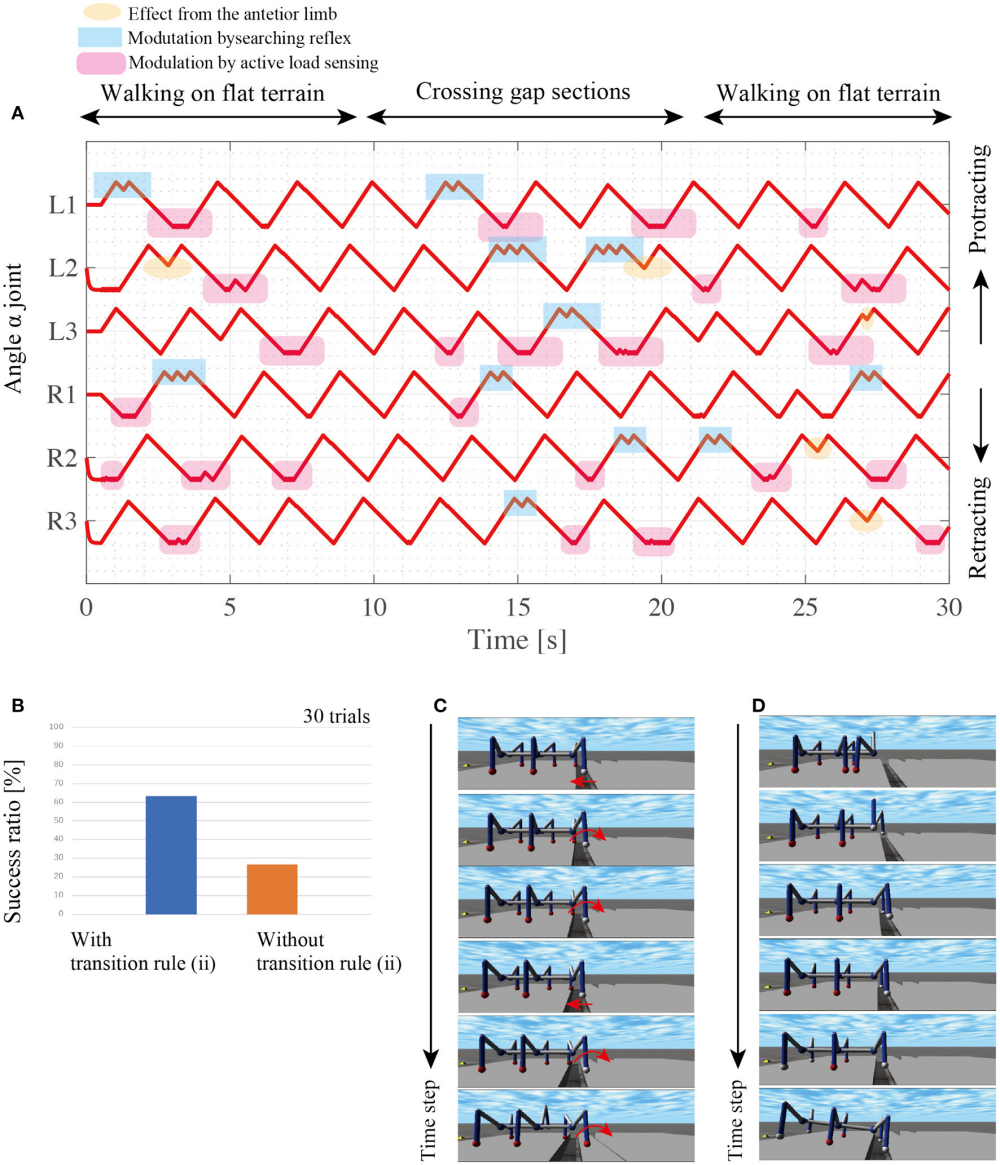
Results of walking experiments on uneven terrain. **(A)** Each history of α joint's angle during crossing gap sections. **(B)** Comparison of success ratios with and without the transition rule (ii). For each control condition, we conducted 30 trials with random initial angles for each limb's α joint. **(C)** Snapshots of successful gap crossing in the trial with transition rule (ii). **(D)** Snapshots of gap crossing failure during walking with transition rule (ii).

During gap crossing, the proposed model modulates the interlimb coordination and resulting in adaptive changes between walking and searching behaviors. At the beginning of the walk with the random, the active load sensing (reflex rule iii) and the effect from the posterior limb (reflex rule iv) modulates the interlimb coordination from the random initial condition to tetrapod gaits as shown in Figure 10. During the gap crossing, several limbs generate searching behaviors depending on the lack of foothold. Note that other limbs adaptively keep their control modes of *stance mode* by using to secure the support polygon by the feedback from the active load sensing. Then, after the gap crossing, all limbs modulate their interlimb coordination for stable locomotor patterns by the fundamental reflex rules. The robot with the searching reflex achieves 60% success ratio, while the robot without the searching reflex achieves a success ratio of under 30% (Figure 10B).

Figure 10C shows snapshots of the successful trial in the gap crossing by the robot with reflex rule (ii). The L1 limb retracts over the gap and does not obtain the foothold. Then, the L1 controller switches the control mode from the stance mode to the swing mode *via* the searching reflex. During the L1 limb's protraction, the robot body moves forward by other limbs' retractions, and consequently, L1 overcomes the gap and obtains a new foothold.

The robot with the searching reflex, however, fails to cross the gap due to stacking behaviors as shown in the snapshots of the failed trial (Figure 10D). In these snapshots, when the L1 and R1 limbs lift, the robot maintains the static support polygon with the L2, L3, R2, and R3 limbs. However, as the supporting limbs retract, the COM moves outside the support polygon, and consequently, the robot loses body balance during searching. Although the proposed model sometimes fails the crossing gap because of no sensory modulation during the stance phase, these results show that the proposed simple interlimb coordination mechanism play a pivotal role for the robot to change its limb behavior between walking step and searching step in response to the lack of footholds around AEP.

## 4. Discussion

The significance of the present study is to demonstrate that insect-like adaptive locomotor patterns (e.g., adaptation to locomotion speed, leg amputation, and gap crossing) can emerge *via* a simple chain of reflex mechanisms. Owing to the simplicity of the proposed model, the series of transition rules can be interpreted as a simple control strategy: each limb tries to create a static support polygon in a decentralized manner. This simple control strategy seems to be reasonable in insect locomotion because the insect's morphology (e.g., low COM due to the sprawled posture and a redundant number of limbs) has great advantages in securing support polygons. While complex neural network models help us to clarify the correspondence between the neural networks in the insect and the structure of neural modules in the modeling studies, the simple model allows us to understand the essences of the underlying control mechanism as well as introduce them to adaptive robot control.

While our model is abstracted, each reflex mechanism is similar to the biological findings. Rule (i) follows the reflex mechanism based on joint angles (Akay et al., 2004; Ekeberg et al., 2004). Rule (ii) and rule (iii) satisfy the physiological findings that sensory input signaling ground contact takes over the effects of command neurons for searching behaviors (Berg et al., 2015). Rule (iv) is similar to the effects from the posterior to the anterior limbs (Borgmann et al., 2009). Although our proposed model does not describe the details of the above sensory feedback mechanisms with interactions among sensor organs (e.g., mechanoreceptors), motor- and intern-neurons, the simple model integrates substantial sensory feedback mechanisms for adaptive interlimb coordination in response to locomotor frequency, leg amputation, and a gap of foothold.

Furthermore, the structure of the proposed model could shed new light on the control mechanism underlying insect adaptive searching behaviors. According to biological experiments, two control schemes, the “two motor patterns hypothesis” and “two control modes hypothesis,” have been proposed (Dürr et al., 2018). In the two motor patterns hypothesis, the control system has two distinct motor patterns for the long step of the walking limb and the short step of the searching limb. Walking and searching behaviors are realized by switching these motor patterns depending on the sensory information. In contrast, the two control modes hypothesis assumes a control mechanism for each swing phase and stance phase, and adaptive walking and searching steps emerge from reasonable switching between the control mechanisms. In this sense, our proposed model agrees with the two control modes hypothesis. Note that our simple model shows the significance of physical interaction in the two control modes hypothesis. Although each limb locally implements two control modes (namely, swing mode and stance mode) and simple reflex rules, the physical interaction with the environment globally affects among limbs and makes each limb free or responsible to support body weight. These interactions should be important for each limb to flexibly generate walking and searching steps as well as secure support polygon when an other limb is searching the foothold.

The failure case in the simulation experiments suggests that flexible coordination between the joints of one limb (e.g., intralimb coordination) is required to improve the adaptability of the proposed model. In the amputated experiments, the robot with amputated L1, R1 limbs cannot secure the support polygon by other limbs, and it tumbles. The simulated robot body model has COM at the middle of the trunk, the PEP limb position of the middle limbs induces the projected COM on the ground to go outside of the support polygon. These failure cases suggest that limbs should change the AEP and PEP position for a stable support polygon. According to insect behaviors, the actual insects modulate the AEP and PEP positions depending on the limb amputation and carrying loads (Delcomyn, 1991; Zollikofer, 1994).

Besides, in the gap crossing experiments, the COM also goes outside of the support polygon during the responsible limb's stance phase, whereas the anterior limbs searching footholds. This is because the target angular velocities of the α joints are set as constant values ω_sw_ and ω_st_, and the supporting limb keeps retracting regardless of the projected COM going outside the support polygon, as shown in Figure 10C. In contrast, actual insect animals modulate their joint angular velocity depending on the situation (Watson et al., 2002). Furthermore, the searching behavior of each limb in simulation moves around the predesigned AEP in the sagittal plane whereas the insects (e.g., stick insect) spread the AEP of the forelimb forward and lateral (Theunissen and Dürr, 2013). These gaps in limb behaviors between the simulation and insect animals suggest that intra-limb coordination should be considered to generate flexible limb motion in both swing and stance modes.

Although we simplified the robot structure, a more insect-like limb structure may induce the robot to exploit physical interaction with the environment. The proposed model exhibits various insect-like gait patterns as shown in Figure 7; however, each limb shows a chattering step at the PEP position. This is because active load sensing is conducted by the limb lifting off the ground. Therefore, if the limb is responsible for the static supporting polygon, once the limb lifts off the ground, the responsible limb touches the ground again, resulting in chattering behaviors. Introducing a flexible foot segment like an insect's tarsus makes it possible to detect the limb's responsibility by sensing the strain of the flexible tip of the foot segment before the limb lifts off completely.

## 5. Conclusion

To elucidate the essential interlimb coordination mechanism underlying adaptive insect's walking and searching behaviors, we developed the simple model that consists of two control models (i.e., swing and stance modes) and four substantial reflex rules. Although the results of the simulation experiments suggest the requirement of additional control mechanisms for flexible intralimb coordination, the robot with the proposed simple interlimb coordination mechanism exhibits various speed-dependent gait patterns, adaptation to leg amputation, and flexible switching between the walking step and searching step during the gap crossing. These results show that simple decentralized control mechanism, e.g., active load sensing, and physical interaction with the environment generate the flexible changes between walking and searching limb behaviors with interlimb coordination for secure support polygons.

For further study, we will develop a physical robot considering the flexibility of the foot segment and evaluate the proposed model in a real-world environment. Furthermore, the intralimb coordination mechanism will be introduced in the proposed model so that each limb can adaptively change its stride speed and foot trajectory depending on the robot morphology and locomotor environments.

## Data Availability Statement

The original contributions presented in the study are included in the article/Supplementary Material, further inquiries can be directed to the corresponding author.

## Author Contributions

AF, TK, RK, and AI conceived the research. AF and WS conducted the experiments and analyzed the results. All authors developed the mathematical modeling and reviewed the manuscript.

## Funding

This work was supported by JSPS KAKENHI [Grant-in-Aid for Scientific Research(S)] grant no. JP17H06150.

## Conflict of Interest

The authors declare that the research was conducted in the absence of any commercial or financial relationships that could be construed as a potential conflict of interest.

## Publisher's Note

All claims expressed in this article are solely those of the authors and do not necessarily represent those of their affiliated organizations, or those of the publisher, the editors and the reviewers. Any product that may be evaluated in this article, or claim that may be made by its manufacturer, is not guaranteed or endorsed by the publisher.

## Supplementary Material

Supplementary Movie 1 Tripod gait in Section 3.1.

Supplementary Movie 2 Tetrapod gait in Section 3.1.

Supplementary Movie 3 Wave gait in Section 3.1.

Supplementary Movie 4 The behavior of the robot with amputated L2 R2 limbs in Section 3.2.

Supplementary Movie 5 Gap crossing experiment in Section 3.3.

Supplementary Movie 6 The searching behavior of the robot with reflex rule (ii) in the successful trials in Section 3.3.

Supplementary Movie 7 The stacking behavior of the robot with reflex rule (ii) in the failed trial in Section 3.3.

## Appendix A: Foot Trajectory

In the stance mode, the limb generates retracting motion to kick the ground with a target angular velocity in the α joint, ${\bar{\dot{\theta }}}_{i,j}^{\alpha }$. More specifically, the target values for each joint are described to generate a specific state foot trajectory as follows:

(6) $$\begin{array}{l} {\bar{\dot{\theta }}}_{i,j}^{\alpha }=\omega_{\text{st}}, \end{array}$$

(7) $$\begin{array}{l} {\bar{\theta }}_{i,j}^{\beta }=\frac{\pi -{\bar{\theta }}_{i,j}^{\gamma }}{2}+\tan^{-1}\left(\frac{H\cos\theta_{i,j}^{\alpha }}{W}\right)-\frac{\pi }{2}, \end{array}$$

(8) $$\begin{array}{l} {\bar{\theta }}_{i,j}^{\gamma }=\cos^{-1}\left(\frac{L_{\text{UPR}}^{2}+L_{\text{BTM}}^{2}-L_{\text{st}}^{2}}{2L_{\text{UPR}}L_{\text{BTM}}}\right), \end{array}$$

where *L*_UPR_ and *L*_UPR_ are the lengths of the upper and bottom links of the limb, respectively, and *L*_st_ is a parameter that reflects the target foot trajectory. The parameter *L*_st_ is calculated as follows:

(9) $$\begin{array}{l} L_{\text{st}}=H^{2}+{\left(W/\cos\theta_{i,j}^{\alpha }\right)}^{2}. \end{array}$$

where *H* and *W* are the target height of the body unit and width of the target foot trajectory, respectively.

In the swing mode, the limb generates a protracting motion along a round trajectory for ground clearance. As in the stance mode, the joint α is controlled to achieve the target angular velocity, where the joints β and β are controlled to achieve target angles. The target values are described as follows:

(10) $$\begin{array}{l} {\bar{\dot{\theta }}}_{i,j}^{\alpha }=\omega_{\text{sw}}, \end{array}$$

(11) $$\begin{array}{l} {\bar{\theta }}_{i,j}^{\beta }=\frac{\pi -{\bar{\theta }}_{i,j}^{\gamma }}{2}-\frac{\pi }{2}, \end{array}$$

(12) $$\begin{array}{l} {\bar{\theta }}_{i,j}^{\gamma }=\cos^{-1}\left(\frac{L_{\text{UPR}}^{2}+L_{\text{BTM}}^{2}-L_{\text{sw}}^{2}}{2L_{\text{UPR}}L_{\text{BTM}}}\right), \end{array}$$

where *L*_sw_ is a parameter that reflects the target foot trajectory. The parameter *L*_sw_ is calculated as follows:

(13) $$\begin{array}{l} L_{\text{sw}}={\left(H-h\left(\theta_{i,j}^{\alpha }\right)\right)}^{2}+{\left(W/\cos\theta_{i,j}^{\alpha }\right)}^{2}, \end{array}$$

(14) $$\begin{array}{l} h\left(\theta_{i,j}^{\alpha }\right)=H\cos\left(\frac{\pi }{\theta_{\text{AEP}}-\theta_{\text{PEP}}}\left(\theta_{i,j}^{\alpha }-\frac{\theta_{\text{AEP}}+\theta_{\text{PEP}}}{2}\right)\right). \end{array}$$

where $h\left(\theta_{i,j}^{\alpha }\right)$ is a function of $\theta_{i,j}^{\alpha }$ for the calculation of the target height of the foot. θ_AEP_ and θ_PEP_ are positive and negative constant values for the angular limitation of joint α at the AEP and PEP, respectively. By switching between the two control modes, the limb generates stride motion along the semicircular trajectory.
